# Morphological and Molecular Characterization of a New *Trichuris* Species (Nematoda- Trichuridae), and Phylogenetic Relationships of *Trichuris* Species of Cricetid Rodents from Argentina

**DOI:** 10.1371/journal.pone.0112069

**Published:** 2014-11-13

**Authors:** María del Rosario Robles, Cristina Cutillas, Carlos Javier Panei, Rocío Callejón

**Affiliations:** 1 Centro de Estudios Parasitológicos y de Vectores (CEPAVE), CCT- CONICET- La Plata/Universidad Nacional de La Plata, La Plata, Buenos Aires, Argentina; 2 Departmento de Microbiología y Parasitología, Facultad de Farmacia, Universidad de Sevilla, Sevilla, España; 3 Virología, Facultad de Ciencias Veterinarias, Universidad Nacional de La Plata, La Plata, Buenos Aires, Argentina; 4 Consejo Nacional de Investigaciones Científicas y Técnicas (CONICET), Buenos Aires, Argentina; INRA, France

## Abstract

Populations of *Trichuris* spp. isolated from six species of sigmodontine rodents from Argentina were analyzed based on morphological characteristics and ITS2 (rDNA) region sequences. Molecular data provided an opportunity to discuss the phylogenetic relationships among the *Trichuris* spp. from Noth and South America (mainly from Argentina). *Trichuris* specimens were identified morphologically as *Trichuris pardinasi*, *T. navonae*, *Trichuris* sp. and *Trichuris* new species, described in this paper. Sequences analyzed by Maximum Parsimony, Maximum Likelihood and Bayesian inference methods showed four main clades corresponding with the four different species regardless of geographical origin and host species. These four species from sigmodontine rodents clustered together and separated from *Trichuris* species isolated from murine and arvicoline rodents (outgroup). Different genetic lineages observed among *Trichuris* species from sigmodontine rodents which supported the proposal of a new species. Moreover, host distribution showed correspondence with the different tribes within the subfamily Sigmodontinae.

## Introduction

Species of *Trichuris* Roederer, 1761 (Nematoda: Trichuridae) have a cosmopolitan distribution and parasitize a broad range of mammalian hosts, such as ruminants, marsupials, rodents, and primates, including humans [1], [2]. The presence of *Trichuris* species among host species is probably related to a combination of factors, such as the immunologic status and the behavior of the host species, and characteristics of the environment where the host lives [2], [3]. To discriminate between the alternative hypotheses of co-speciation (host-parasite) versus geographical differentiation, it is necessary to integrate studies of both morphological and molecular analysis [4].

Several features, such as the presence/absence of the spicular tube, the shape and distribution of the spines of the spicular sheath, length of the spicule and the cloacal tube, the shape of the proximal and distal cloacal tube, and the vulvar morphology, along with classic morphometric characteristics have been used as characteristics with high discriminatory value to differenciate the species of *Trichuris* i.e. [5]–[10]. Moreover, scanning electron microscopy (SEM) has been used as a diagnostic tool in some studies [10]–[15]. *Trichuris* species have been described with a narrow range of anatomic and biometric characteristics; and they have been insufficiently compared with their congeneric species i.e. [5], [16]–[19]. Consequently, different populations with overlapping morphometrical features [8] result in taxonomic and nomenclatorial problems, e.g. individuals of the same species recognized as different species (under different names: synonyms), and different species referred as the same species (sibling species) [20], [21].

To date, 24 *Trichuris* species have been described from 10 families of North and South American rodents [22]. Among these, three species are parasites of Cricetidae in North America: *T. opaca* Barker and Noyes, 1915 from Arvicolinae, *T. neotomae* Chandler, 1945 and *T. peromysci* Chandler, 1946 from Neotominae; and five parasites of Cricetidae in South America: *T. chilensis* Babero, Cattan and Cabello, 1976, *T. travassosi* Correa Gomes, Lanfredi, Pinto and Souza, 1992, *T. laevitestis* Suriano and Navone, 1994; *T. pardinasi* Robles, Navone and Notarnicola, 2006, and *T. navonae* Robles, all from Sigmodontinae. The last three species were found from Argentina [7], [10], [22].

Of the *Trichuris* from Cricetidae, 66% share a general morphological pattern, including the absence of a spicular tube, spicular sheath with spines (most with a cylindrical shape), and a non-protrusive vulva, thus these can be separated mainly by morphometric characters with high discriminatory value [6], [10], [22]–[25]. However, this also exemplifies the difficulty in finding morphological differences among species in this genus. For this reason, some studies have used isoenzymatic patterns and molecular studies to identify these nematodes [25]–[29]. The genes encoding the rRNA subunits are particularly useful in phylogenetic studies. In fact, recent studies have demonstrated that internal transcribed spacers (ITS1-5.8S-ITS2) of nuclear ribosomal DNA (rDNA) provide genetic markers for the accurate identification of closely related nematode species [30]. Sequence data of the ribosomal ITS2 have also been shown to be a valuable tool in species identification; first because they are highly species-specific and, second because they are flanked by conservative regions of the rDNA that allows the use of universal primers that bind to the 5.8S and 28S rDNA genes of many helminths e.g. [31]–[37]. Since, the ITS2 region is much more variable than ITS1, it allows a better discrimination at the species level. For example, ITS2 have been used for the unequivocal delineation of morphologically well defined adults such as *Trichuris ovis*, *Trichuris leporis* and *Trichuris suis* with high levels (32.8–58.64%) of interspecific variation e.g. [21], [27]. Also, ITS2 provides significant phylogenetic insights [38], [39]. Studies on the comparative phylogeny of taxa strongly linked by an ecological factor, such as parasitism, have shown that the degree of phylogenetic congruence increases with the forced character of the host–parasite relationship [40].

The purpose of this paper was to study different populations of *Trichuris* isolated from six species of Sigmodontinae rodents from Argentina, based on morphological characteristics and ITS2 of nuclear rDNA region sequences. Also, the description of a new species of *Trichuris* is provided in this study, and the level of variation among the ITS2 sequences of studied populations was determined. Molecular data are also used to analyze and discuss the phylogenetic relationships among the *Trichuris* spp from the Americas, and mainly from Argentina.

## Material and Methods

Cricetid rodents were trapped during different field studies between 2009 and 2012 (see collectors in acknowledgements). A total of 81 adult specimens of *Trichuris* were studied from six species of Sigmodontinae rodents (Cricetidae) from eight/seven localities respectively: 61 for morphological analyses and 20 for molecular characterization (see Table 1).

**Table 1:** *Trichuris* specimens studied from different rodent species of Argentina.

*Trichuris* spp. Number of studied specimens		Host species	Locality/Province	Code	Geographical point
*Morphological*	*Molecular*				
8	3	*Phyllotis bonariensis*	Cerro Bahía Blanca, Parque Provincial Ernesto Tornquist, Sierra de la Ventana, Partido de Tornquist, Buenos Aires province	SV	38°04′47.99″ S, 62°00′22.48″ W
10	2	*Phyllotis xanthopygus*	Cerro Los Linderos, Departamento Calamuchita, Córdoba province	SC	32°00′17.82″ S, 64°56′ 01.51″ W
Robles, 2011; Robles and Navone, 2014	4	*Akodon montensis*	Refugio Moconá, Departamento San Pedro, Misiones province	RM	27°8′ S, 53°55′ W
10	3		Reserva de Vida Silvestre Urugua-í, Fundación Vida Silvestre, Departamento General Manuel Belgrano, Misiones province	UR	25°59′08.19″ S, 54°06′36.15″ W
6	1		Campo Anexo M. Belgrano, INTA, San Antonio, Departamento General Manuel Belgrano, Misiones province	SA	26°02′52.60″ S, 53°46′21″ W
2	1	*Thaptomys nigrita*	Reserva de Vida Silvestre Urugua-í, Fundación Vida Silvestre, Departamento General Manuel Belgrano, Misiones province	UR	25°58′32.29″ S, 54°07′00.08″ W
3	2		Campo Anexo M. Belgrano, INTA, San Antonio, Departamento General Manuel Belgrano, Misiones province	SA	26°02′54.21″ S, 53°46′32.40″ W
5	1	*Necromys obscurus*	Estación Experimental Agropecuaria Balcarce, INTA, Partido de Balcarce, Buenos Aires province	BA	37°42′59.53″S, 58°16′23.12″W
12	2	*Sooretamys angouya*	Refugio Moconá, Departamento San Pedro, Misiones province	RM	27°8′ S, 53°55′ W
3	1		Estación de Animales Silvestres Guaycolec, Departmento Formosa, Formosa Province	GU	25°58′51″ S, 58°9′52″ W
2	0		Reserva de Usos Múltiples Guaraní, Departamento Guaraní, Misiones province	RG	26°56′ S, 54°13′ W

### Ethics Statement

The research has been conducted according to Argentine laws. Sample collection was carried out during fieldwork under oficial permits granted by Ministerio de Asuntos Agrarios de la provincia de Buenos Aires (expedient 22500-7981-2010-0) and Organismo Provincial para el Desarrollo Sustentable (OPDS) (expedient 2145-6077/10), Ministerio de Producción y Ambiente de la Provincia de Formosa (authorization s/n; Guía de Tránsito: 004076), Ministerio de Ecología, RNR y Turismo, Provincia de Misiones (authorization #27, Guía Tránsito 000316). This study was carried out in accordance with the recommendations in the Guide for the Care and Use of Laboratory Animals of the National Institutes of Health. The specimens obtained with methods for live capture were studied and humanely sacrificed (euthanasia by thoracic compression under ether anesthesia), following the procedures and protocols approved by national laws (Animal Protection National law 14.346 and references in the provincial permits) and Ethics Committee for Research on Laboratory Animals, Farm and Obtained from Nature of National Council of Scientific and Technical Research (CONICET) (Resolution 1047, section 2, annex II), and subsequently by National Agency for the Promotion of Science and Technology of Argentina (ANPCYT) (PICT 2010-0924). No endangered species were involved in this study.

### Morphological analysis

Nematodes were preserved in 70% ethanol, and cleared in lactophenol, and studied using a light microscope. Morphological identification was performed using characteristics listed by Robles et al. [10] and Robles [22]. Drawings of specimens of *Trichuris* from *Sooretamys angouya* were made with the aid of a drawing tube. Four specimens of this population were dehydrated in an ethanol series (75%, 80%, 85%, 90%, 96%, 100%), dried using the critical point method, and examined with the aid of a scanning electron microscope (Jeol 6360 LVLV, Tokyo, Japan). Measurements of new species are presented as follows: holotype male or allotype female, and paratypes with mean, standard deviations, and range in parentheses. We tested for statistical differences for some variables. When data met parametric requirements, Student's *t-test* was used for pairwise comparisons; otherwise a non-parametric Mann–Whitney *U-test* was used. For all calculations, we tested significance at the α = 0.05 level. Statistical analysis was performed using Past 3.01 (Paleontological Statistics, free software). All measurements are given in millimeters (mm). The scales of figures are given in micrometers (µm).

### Nomenclatural acts

The electronic edition of this article conforms to the requirements of the amended International Code of Zoological Nomenclature, and hence the new names contained herein are available under that Code from the electronic edition of this article. This published work and the nomenclatural acts it contains have been registered in ZooBank, the online registration system for the ICZN. The ZooBank LSIDs (Life Science Identifiers) can be resolved and the associated information viewed through any standard web browser by appending the LSID to the prefix “http://zoobank.org/”. The LSID for this publication is: urn:lsid:zoobank.org:pub: A22989C7-144F-4F9F-A3E8-0D13023B0413. The electronic edition of this work was published in a journal with an ISSN, and has been archived and is available from the following digital repositories: PubMed Central and LOCKSS.

Specimens of nematodes were deposited in the Helminthological Collection of Museo de La Plata (MLP), La Plata, Buenos Aires, and hosts in Mastozoological Collections of the Centro Nacional Patagónico (CNP), Puerto Madryn, Chubut, and Mastozoological Collections of Museo de La Plata (MLP) La Plata, Buenos Aires, Argentina.

### Molecular analysis

The specimens previously identified were washed extensively in 0.9% saline solution and stored in 70% ethanol until used for PCR and sequencing.

#### PCR and sequencing of specimens

Genomic DNA from individual worms was extracted using the DNeasy Blood and Tissue Kit (Qiagen) according to the manufacturer's protocol. Quality of extractions was assessed using 0.8% agarose gel electrophoresis and ethidium bromide staining. The Internal Transcribed Spacer 2 (ITS2) of ribosomal DNA (rDNA) region was amplified by PCR using a Perkin Elmer thermocycler and the following PCR mix: 10 µl 10×PCR buffer, 2 µl 10 mM dNTP mixture (0.2 mM each), 3 µl 50 mM MgCl_2_, 5 µl primer mix (0.5 mM each), 5 µl template DNA, 0.5 µl *Taq* DNA polymerase (2.5 units) and autoclaved distilled water to 100 µl. The following conditions were applied: 94°C for 3 min (denaturing), 35 cycles at 94°C for 1 min (denaturing), 50°C for 1 min (annealing), 72°C for 1 min (primer extension), followed by 10 min at 72°C. DNA sequences of the forward primer 5.8S F (5′-GTAGGTGAACCTGCGGAAGGATCATT-3′) and reverse primer ITS2R (5′-TTAGTTTCTTTTCCTCCGCT-3′) corresponded to the conserved 3′- 5′ ends of the ITS1-5.8S-ITS2 flanking the 5.8S and 28S gene regions. Thus, DNA sequence of the reverse primer was cited by Gasser et al. [41], while forward primer was designed by us. For each set of PCR reactions and extraction of the DNA, samples without DNA (negative) and a known (positive) control DNA samples were also included. The PCR products were checked on ethidium bromide-stained 2% Tris-Borate-EDTA (TBE) agarose gels. Bands were eluted from the agarose by using the Wizard SV Gel and PCR Clean-Up System (Promega). The purificated PCR products were concentrated, and directly sequenced by Stab Vida (Portugal). The rDNA intra-individual similarity was determined by sequencing between three to five clones of one individual per population of *Trichuris* species. Thus, the isolated DNA was cloned into *Escherichia coli* DH5α using pGEM-T Easy vector system (Promega). Transformed cells were selected by overnight incubation at 37°C on LBB/Amp/X-gal/IPTG plates. In order to check for successful cloning and to study the intra-individual variation, at least ten single recombinants (clones) were screened for the DNA insert and sequenced. The ten clones containing the correct insert were used to inoculate 5 ml of LBB/Amp broth and incubated, shaked at 37°C for 12 h. Plasmid were purified using a Wizard Plus SV (Promega)and sequenced by Stab Vida (Portugal) with an universal primer (M13). The intra-specific similarity was determined for the rDNA by sequencing, at least, three individuals of each locality and host. Furthermore, all the sequences were aligned and compared with each other using the CLUSTAL W program. Alignments were manually adjusted.

Restriction maps of the different ITS2 sequences were determined by using the “Map” program available on Gen Bank.

#### Sequence analysis

Phylogenetic trees based on ITS2 rDNA were rooted by including five outgroups representing members of the genus *Trichuris* from Murinae and Arvicolinae rodents isolated from Europe, Africa and North America (Table 2). Phylogenetic trees were produced using three methods: Maximum Likelihood (ML), Maximum Parsimony (MP) and Bayesian Inference (BI), using the PhyML package [42], MEGA 5.0 program [43] and MrBayes version 3.1.2 [44], respectively. jModeltest version 0.1.1 [45] was used to choose a best-fit model of sequence evolution [45]. For the Bayesian analysis, we ran three independent runs of four Markov chains for 10 million generations, sampling every 500 generations. For ML inference, the rapid bootstrap algorithm (with GTRCAT) was used (1000 replicates) to assess the relative reliability of clades, whereas the best ML tree was found using the GTRGAMMA model and a more thorough optimization.

**Table 2:** *Trichuris* species included in the phylogenetic analysis based on the ITS2 rDNA.

Species	Host species	Host family/subfamily	Geographical Origin	Code	Accession Number
*Trichuris* sp.	*Microtus townsendii*	Cricetidae/Arvicolinae	Oregon, USA	OR	FR849676
*Trichuris arvicolae*	*Myodes glareolus*	Cricetidae/Arvicolinae	Montseny, Spain	MO	FR849660
*Trichuris muris*	*Mus domesticus*	Muridae/Murinae	Calafel, Spain	CA	FN543175
*Trichuris carlieri*	*Gerbilliscus vicinus*	Muridae/Murinae	Maguha, Tanzania	MA	JX683522
*Trichuris mastomysi*	*Mastomys natalensis*	Muridae/Murinae	Berega Tanzania	BE	JX683517

## Results

### Morphological analysis

Specimens of *Trichuris* from eight populations were identified based on morphological characteristics (Table 3). The specimens found in *Phyllotis bonariensis* Crespo, 1964 and *P. xanthopygus* Waterhouse, 1837 were identified as *Trichuris pardinasi*; and those found in *Akodon montensis* Thomas, 1913 and *Thaptomys nigrita* (Lichtenstein, 1829) as *T. navonae*. Although the *Trichuris* specimens from *Necromys obscurus* (Waterhouse, 1837) were studied in detail, species identification was not possible since only one male was present. A new species of *Trichuris* was found in *Sooretamys angouya* (Fischer, 1814), which is described here.

**Table 3:** *Trichuris* species found from different rodent species from Argentina (see Table 1), with new localities records and molecular data.

Species	Host species	Localities	Base pairs	G+C%	GenBank Accession number
*T. pardinasi*	*Phyllotis bonariensis*	SV	429 430 431	60.3 60.7 61.7	HG934448 HG934445 HG934449
	*Phyllotis xanthopygus*	SC (new locality)	429 433	60.4 60.1	HG934447 HG934446
*T. navonae*	*Akodon montensis*	RM	427 427 427 427	59.4 59.7 60.2 60.1	HG934435 HG934436 HG934437 HG934438
		UR (new locality)	428 435 427	59.6 59.5 59.7	HG934443 HG934444 HG934441
		SA (new locality)	427	59.7	HG934434
*T. navonae*	*Thaptomys nigrita*	UR (new locality)	427	59.7	HG934439
		SA (new locality)	427 427	59.5 60.4	HG934440
*Trichuris* sp.	*Necromys obscurus*	BA (new locality)	388	59.3	HG934450
*Trichuris bainae* n. sp	*Sooretamys angouya*	RM	441 441	59.9 59.7	HG934431 HG934432
		GU (new locality)	441	60.6	HG934433

Moreover, Cerro Los Linderos (SC), Córdoba province; Reserva de Vida Silvestre Urugua-í (UR) and Campo Anexo M. Belgrano, INTA, San Antonio (SA), Misiones province; Estación Experimental Agropecuaria Balcarce, INTA (BA), Buenos Aires province, and Estación de Animales Silvestres Guaycolec (GU), Formosa Province constitute new geographical records for the genus *Trichuris* (Table 3).

#### Description

***Trichuris bainae*** **n. sp. urn: lsid: zoobank.org: act: C53665A4-4DAE-4A2C-988A-59754EBA54A7.**

(Figs. 1, 2)

**Figure 1:**
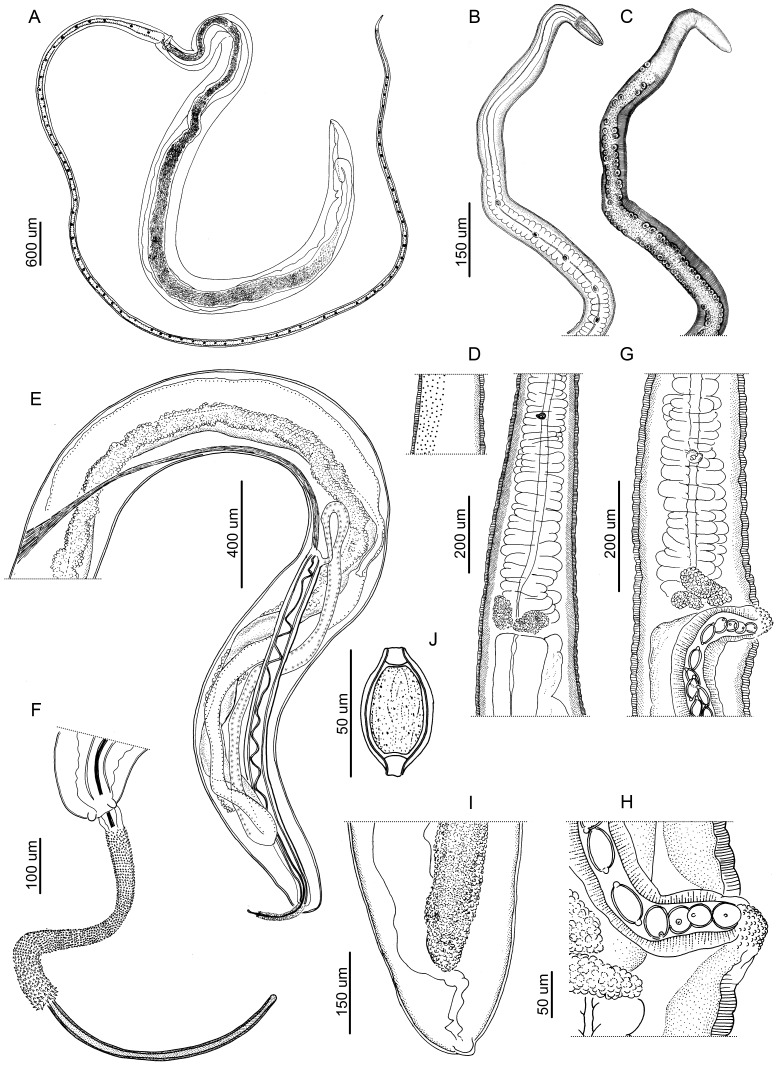
Drawings of *Trichuris bainae* n. sp. (A) Complete female specimen. (B) Esophagus, muscular and stichosome portions. (C) Esophagus, muscular and stichosome portions, with bacillary band and cuticular inflations view. (D) Male, esophagus-intestine junction and proximal portion of testis, with bacillary band view. (E) Male, posterior end, spiny spicular sheath, spicule and proximal and distal cloacal tube, lateral view. (F) Male, detail of the posterior extremity, lateral view. (G) Female, esophagus-intestine junction and vulva, lateral view. (H) Female, detail of vulva, lateral view. (I) Female, posterior end, lateral view. (J) Egg.

**Figure 2:**
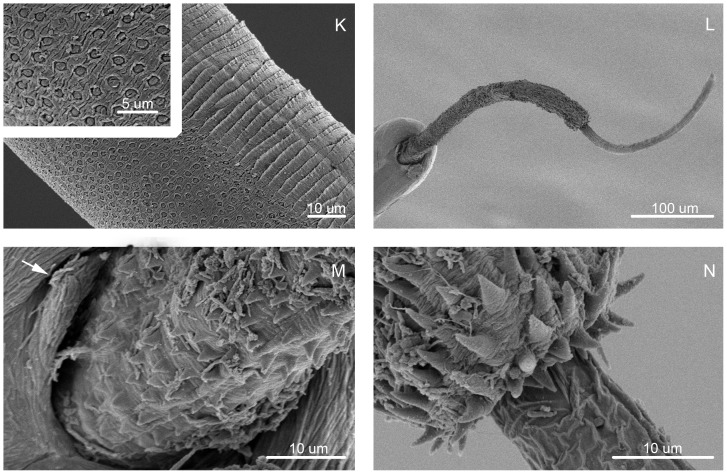
Scanning electron micrographs of *Trichuris bainae* n. sp. - SEM. (K) Bacillary band, with detail of bacillary glands. (L) Male, posterior end, ventral view. (M) Male, detail of the proximal portion of spiny spicular sheath. (N) Male, detail of the distal portion of spiny spicular sheath.

*Diagnosis*: Cuticle with fine transversal striation (Figs. 1C, 2K). Anterior part of body long, narrow, tapered, and whip-like; posterior part of body broad, and handle-like (Fig. 1A). Ratio between anterior and posterior body length is 1∶1.8 in males and 1∶1.4 females. Stichosome with 1 row of stichocytes, and 1 pair of conspicuous cells at esophagus-intestinal junction level (Figs. 1B, 1D, 1G). Male with spicular tube absent. Proximal cloacal tube, united laterally to distal cloacal tube (Fig. 1E). Spicular sheath cylindrical with spines distributed from proximal to distal portion; distal spines very sharpened and joined together (Figs. 1F, 2L, 2M, 2N). Testis ends near final third of distal cloacal tube, showing different degree of convolutions (Fig. 1E). Cloaca subterminal with 1 pair of paracloacal papillae, not ornamented (Figs. 1F, 2M). Female with ornamentated protrusive vulva located at esophagus-intestinal junction level (Figs. 1G, 1H). Anus subterminal, with long caudal end finished with terminal torsion (Fig. 1I).

Bacillary band located laterally in anterior portion of body (Figs. 1C, 2K). Bacillary band 0.05–0.09 from anterior end of body, and extends to body width region of 0.18–0.24. With SEM, cuticular inflations appear bordering bacillary band from 0.2–0.37 to 0.6–0.87 in the anterior end of body, forming low rings of thick walls and very reduced interior cavity. These structures limit laterally to abundant and visible bacillary glands with conspicuous pore (Fig. 1C, 2K). Cuticle around vulvar aperture with transversally striated pattern (Fig. 1H).

*Male (6 specimens):* Body length 13.9, 13.6±0.58 (12.97–14.4). Anterior portion of body 8.67, 8.98±1.04 (7.4–9.92) long and thick portion of body 5.25, 4.96±0.83 (4.15–6.05) long. Anterior body width 0.75, 0.068±0.01 (0.050–0.075), maximum posterior body width 0.25, 0.35±0.061 (0.27–0.42), width at esophagus-intestinal junction level 0.17, 0.22±0.035 (0.17–0.25) (Figs. 1B, 1D). Total length of esophagus 8.65, 8.89±0.89 (7.3–9.9), muscular portion 0.45, 0.36±0.16 (0.2–0.47) long, stichosome portion 8.2, 8.7±1.1 (7–9.8) long. Spicule length 1.94, 2.16±0.13 (1.93–2.3) (Fig. 1E). Spicular sheath densely spinose 1.7, 1.8±1.1 (1.5–2) long (Figs. 1F, 2L). Proximal cloacal tube 1.2, 1.57±0.35 (1.19–2.11) long, distal cloacal tube 1.79, 1.36±0.31 (1.15–1.9) long (Fig. 1E). Ratio between total body length and posterior portion length 2.64, 2.89±0.45 (2.22–3.23). Ratio between total body length and spicule length 7.16, 6.33±0.53 (5.87–6.97). Ratio between posterior portion length and spicule length 2.7, 2.32±0.52 (1.89–3.13). Ratio beween proximal cloacal tube length and distal cloacal tube length 0.67, 1.15±0.17 (1.01–1.43). Ratio between maximum posterior body width and posterior portion length 0.04, 0.07±0.01 (0.06–0.095).

*Female (2 specimens):* Body length 22.18, 23.3. Anterior portion of body 12.66, 14.2 long and thick portion of body 9.52, 9.1 long (Fig. 1A). Anterior body width 0.05, 0.075, maximum posterior body width 0.5, 0.5; width at esophagus-intestinal junction 0.2, 0.22 (Fig. 1G). Total length of esophagus 12.65, 13.7, muscular portion 0.38, 0.35 long, stichosome portion 12.27, 13.66 long. Distance between esophagus-intestinal junction and vulva 0.22, 0.20. Eggs oval, with bipolar plugs, (n = 10) 0.020–0.025×0.045–0.05 (Fig. 1J). Ratio between total body length and posterior portion length 2.33, 2.56. Ratio between maximum posterior body width and posterior portion length 0.052, 0.055.

#### Taxonomic summary

*Type host species*: *Sooretamys angouya* (Fischer, 1814) (Sigmodontinae: Oryzomyini). *Symbiotype*: Female CNP 1998. *Other hosts housed*: CNP 2529 and CNP 3634.

*Type locality:* Refugio Moconá (27°8′ S, 53°55′ W), Guaraní Department, Misiones province, Argentina.

*Other localities*: Estación de Animales Silvestres Guaycolec (25°98′ S, 58°16′ W), Formosa Department, Formosa Province and Reserva de Usos Múltiples Guaraní (26°56′ S, 54°13′ W), Guaraní Department, Misiones province.

*Site of infection*: Caecum.

*Type specimens:* Holotype male MLP-He 6760, allotype female MLP-He 6761, 6 paratypes MLP-He 6762 deposited at the Helminthological Collection of the Museo de La Plata.

*Etymology:* Dedicated to the memory of Odile Bain, a widely recognized parasitologist from Paris, France; who contributed valuable knowledge on trichurid nematodes from many host groups and different parts of the world.

#### Differential diagnosis

The *Trichuris* species from North and South American rodents were compared by different morphometric features [5]–[7], [9], [10], [19], [22]–[24], [46]–[54]. *Trichuris bainae* n. sp. resembles *T. travassosi* and *T. navonae* in their similar general size, cloacal tubes and distance from anterior end to vulva. However, the new species differs from *T. travassosi* by the unequal distribution of the spines on the spicular sheath, and differs from both species by the presence of a vulva ornamented with spines as well as morphometric features.

*Trichuris bainae* n. sp. can be separated from eight of the species that parasitize American rodents, i.e., *T. citelli*, *T. perognathi*, *T. neotomae*, *T peromysci*, *T. madisonensis*, *T. dipodomys*, *T. fulvi* and *T. laevitestis* by the absence of a spicular tube (the spicule lies entirely within the distal cloacal tube).

The new species differs from *T. opaca*, *T. fossor*, *T. citelli*, *T. neotomae*, *T. dipodomys*, and *T. bursacaudata* by lacking a spicular sheath with a spiny distal spherical bulge or a spiny campanuliform shape. Among those species with a cylindric spicular sheath, the new species can be separated from *T. travassosi* and *T. pampeana* by the distribution of the spines.

The new species has a shorter spicule than T. myocastoris, T. bradleyi, T. chilensis, T. fulvi, T. robusti, T. laevitestis, T. bursacaudata, T. pampeana and T. pardinasi and longer than T. opaca, T. fossor, T. perognathi, T. neotomae, T peromysci, T. madisonensis, T. dipodomys, and T. elatoris. Although the ranges of spicule length among T. bainae n. sp., T. travassosi and T. navonae overlap in part, the means are differents (2.16, 1.63 and 2.3, respectively).

Moreover, T. bainae n. sp. has a shorter distal cloacal tube than T. bradleyi, T. chilensis, T. robusti, T. bursacaudata, T. pampeana, and T. pardinasi and longer than T. perognathi, T. neotomae, T. peromysci, T. dipodomys and T. fulvi.

The most similar biometrical features were found between *T. bainae* n. sp. and *T. navonae*. However, some statistical differences were found in characters such as spicule length (*T*-test, *t* = 6.61, *p*<0.001), proximal cloacal tube (*T*-test, *t* = 3.94, *p*<0.001), and the ratio between posterior portion length and spicule length (*U*-test, w = 12, *p* = 0.037).

The new species has an ornamentated protrusive vulva, which is absent in the rest of the species from American rodent hosts. In addition, *T. bainae* n. sp. has a smaller distance to vulva from the anterior end than *T. gracilis, T. opaca, T. myocastoris, T. citelli*, *T. perognathi, T. neotomae, T. peromysci, T. madisonensis, T. dolichotis*, *T. dipodomys, T. bradleyi*, *T. bursacaudata, T. pampeana*, and *T. pardinasi.*

Since the males of *T. gracilis* and *T. dolichotis* have not been described, these species were not included in the preceding comparison. However, the females of these species can be separated from the new species by the dimensions of the body length, and the lengths of the anterior and posterior portions of the body.

### Molecular analysis

Internal Transcribed Spacer 2 (ITS2) rDNA sequences of the specimens of *Trichuris* from seven populations of four species were obtained and analyzed. These sequences ranged from 388 to 441 base pairs (bp) (exclusive of the primers) and their G+C content ranged from 59.3%–61.7% (Table 3).

#### Multiple alignment and sequence model selection

The alignment of 25 ITS2 sequences of *Trichuris* species from rodents from Africa, North America, Europe (Table 2) and South America yield a dataset of 450 characters. jModelTest determined that the best-fit model for ITS2 rDNA datasets was GTR+I+G, which was used for Maximum Likelihood and Bayesian inference.

#### Intra-individual, intra- and inter-specific similarities of *Trichuris* spp. from Argentina based on ITS2 Rdna

The intra-individual similarity, observed for 3 to 5 clones of one individual per population, ranged from 97.4% to 100% (data not shown). The highest value corresponded to all the individuals of *Trichuris* populations from Argentina; nevertheless, the minimum value was observed in *T. navonae* individuals.

The range of intra-specific similarity of *Trichuris* species based on ITS2 rDNA ranged from 95.6% to100%. *Trichuris pardinasi* from Córdoba showed the minimum value (Table 4).

**Table 4:** Intra-specific (*) and inter-specific similarity observed in ITS2 sequences in *Trichuris* populations isolated from different rodent species.

Species	*T. pardinasi* (Buenos Aires)	*T. pardinasi* (Córdoba)	*T. navonae* (Misiones)	*Trichuris* sp. (Buenos Aires)	*T. bainae* n. sp. (Misiones)	*T. bainae* n. sp. (Formosa)
***T. pardinasi*** ** (Buenos Aires)**	95.6–97.3% *					
***T. pardinasi*** ** (Córdoba)**	97.6% *	98% *				
***T. navonae*** ** (Misiones)**	**89.3%**	**90.6%**	96.3%–100% *			
***Trichuris*** ** sp. (Buenos Aires)**	**92.1%**	**92.7%**	**88.8%**	100% *		
***T. bainae*** ** n. sp. (Misiones)**	**88.3%**	**89.8%**	**95.5%**	**88.8%**	97.4%–100% *	
***T. bainae*** ** n. sp. (Formosa)**	**89.7%**	**90.1%**	**94.5%**	**89%**	99.2% *	98.1% *

In order to analyze inter-specific similarities, different species of *Trichuris* isolated from Sigmodontine rodents from Argentina were compared. All the ITS2 rDNA sequences obtained for each species were included in the analysis (alignment not shown). The highest similarity was found between *Trichuris navonae* and *T. bainae* n. sp. both from Misiones (95.5%) and between *T. navonae* and *T. bainae* n. sp. (94.5%), from Misiones and Formosa, respectively. The lowest similarities were found between *Trichuris pardinasi* from Buenos Aires and *T. bainae* n. sp. from Misiones (88.3%), and *Trichuris* sp. from Buenos Aires and *T. navonae* and *T. bainae* n. sp. from Misiones (88.8%) (Table 4).

#### Relationships of *Trichuris* based on ITS2 rDNA sequences

**Figure 3:**
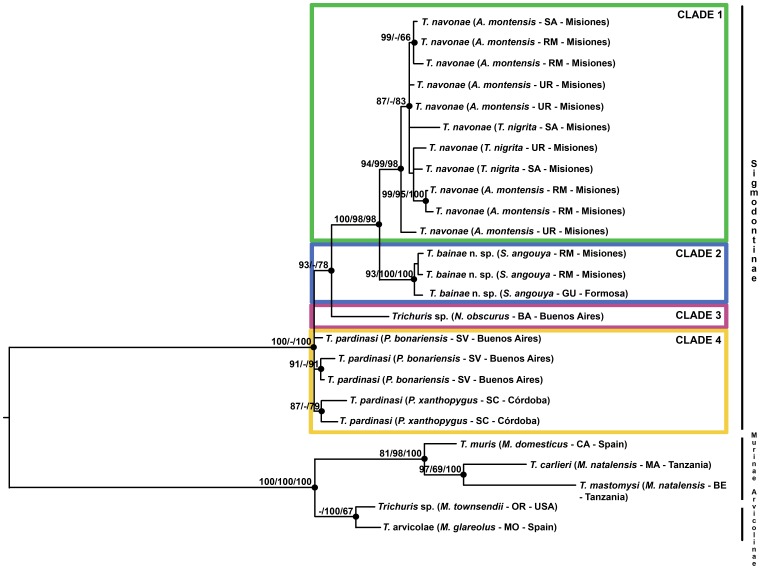
Phylogenetic tree of of *Trichuris* species from rodents of Sigmodontinae, Arvicolinae and Murinae of different geographical origins (see **Table 1** and **2**) based on Internal Transcribed Spacer 2 (ITS2) of ribosomal DNA inferred using Maximum Composite Likelihood. The percentage of replicate trees in which the associated taxa clustered together in the bootstrap test (1000 replicates) is shown on the branches (Maximum Composite Likelihood/Maximum Parsimony/Bayesian Inference). Bootstrap values lower than 65% are not shown.

Phylogenetic trees (Fig. 3) provided robust phylogenetic resolution among most *Trichuris* taxa regardless of the inference method. The topology among *Trichuris* species showed that all the species of *Trichuris* from Argentina are separated from those belonging to *Trichuris* isolated from rodents of Africa, North America and Europe (Bootstrap Values (BP), 100% and 100% in ML and BI methods) (Fig. 3). Furthermore, *Trichuris muris* from Europe and South Africa clustered together and separated of *T. arvicolae* from Europe and North America with high BP (81%, 98% and 100% in ML, MP, and BI respectively) (Fig. 3).

Phylogenetic trees based on ITS2 sequences of *Trichuris* species from Argentina showed four main clades by ML, MP and BI methods (Fig. 3). These four main clades included: Clade 1 clustered *T. navonae* from different hosts from different localities of Misiones region (BP 100%, 98% and 98% in ML, MP and BI methods). Within clade 1, we observed a polytomy of populations of *T. navonae* regardless of the geographical origin and host species. Clade 2 clustered *Trichuris bainae* n. sp., from Formosa and Misiones provinces with high BP values (93%, 100% and 100% in ML, MP and BI). Clade 3 included *Trichuris* sp. from *N. obscurus* from Buenos Aires. Finally, Clade 4 clustered *T. pardinasi* populations from Buenos Aires and Córdoba.

Based on the ITS2 sequences, restriction mapping identified many endonucleases that could be used to delineate different species of *Trichuris* from sigmodontine rodents. Thus, *BsePI*, *BssHII*, *HinfI*, *SacII* and *SstII* sites were present in the sequences of *T. bainae* n. sp., but not in *T. navonae*. Otherwise, *AgeI*, *BshTI, HindIII* and *Sspl* sites were present in the sequences of *T. navonae* but not in those of *T. bainae* n. sp. Interestingly, *HinfI* site was only present in *T. bainae* n. sp., but not in *T. navonae, T. pardinasi* and *Trichuris* sp, therefore this endonuclease is specific for the determination of this new species of *Trichuris*.

## Discussion

The four species of *Trichuris* studied showed the same general morphological pattern in the male reproductive system. In fact, identification of closely related species is very difficult. This is due in part to the phenotypic plasticity of the organisms themselves, host-induced variation, the paucity of morphological features, and the extensive overlap in morphometric characteristics that occur among species e.g. [8], [18], [15], [22]. However, isoenzymatic patterns and molecular studies for identification of these nematodes have been used successfully [25]–[29].

The specimens of *Trichuris* collected from *Sooretamys angouya* in Misiones province belong to a new species with clear morpho-biometrical differences in respect to the rest of the species of *Trichuris*. Molecular data corroborated these results. Although *Trichuris* specimens from *Necromys obscurus* were morphologically studied, species identification was not attained. However, molecular characterization was achieved for those individuals as well as *T. pardinasi* and *T. navonae*. This is the first study that provides the molecular characterization of *Trichuris* species of Sigmodontinae rodents. Also, it is confirmed that *Thaptomys nigrita* is a host of *T. navonae*, since this was previously characterized as *Trichuris* cf. *navonae* [3]. In addition, five new localities for the four species of *Trichuris* studied were recorded.

The internal transcribed spacers (ITS1 and ITS2) located in the ribosomal DNA are considered appropriate molecular markers to resolve relationships at the species level [30]. It has been demonstrated that there is little, if any, intraspecific variation in the sequence of ITS2 and, further, that closely related species show unequivocal differences in these sequences [31]–[35], [55], [56]. In this context, for example, ITS rDNA and 5.8S sequences data have been used to test the existence of two species: *T. muris* and *T. arvicolae* in Muridae and Arvicolidae hosts, respectively and a phylogenetic analysis based on combined 5.8S and ITS2 sequences was carried out [27]. The results obtained clearly indicated that the ITS+ region of rDNA provides genetic markers for whipworm species. Previously, the results of the analysis of the ITS1-5.8S-ITS2 sequence of the ribosomal DNA had confirmed the presence of DNA polymorphisms among *T. muris* isolates from Europe, suggesting the presence of different lineages/species [57].

The percentage of interspecific variation observed among the four species far exceeded the intraindividual and intraspecific variation which is, in general, slight in the ITS2 sequences. These results indicate that *T. pardinasi*, *T. navonae*, *Trichuris* sp. and *T. bainae* n. sp. must be considered different species.

Also, based on ITS2 sequences, some specific recognition sites for endonucleases were detected. *Trichuris navonae* and *T. bainae* n. sp. from Misiones and *T. navonae* from Misiones and *T. bainae* n. sp., from Formosa were the populations with the most similar sequences, but the *HinfI* restriction site was only present in *T. bainae* n. sp., being a useful endonuclease for the determination of this new species.

Phylogenetic studies that includes ecological and host geographical distribution data allows a better interpretation of possible processes that determine the geographical distribution of parasites, subdivisions of populations, speciation events or ecological adaptation [58]. The phylogeographic study of *Trichuris* populations isolated from Cricetid rodents by Callejón et al. [4] used the ITS1-5.8S-ITS2 fragment of the ribosomal DNA and the first subunit of the cytochrome *c* oxidase (*cox1*) region of mitochondrial DNA. This study confirmed the presence of DNA polimorphism among *Trichuris arvicolae* and *Trichuris* sp. isolated from the Western Nearctic and the Western half of the Palearctic region. Also, this survey indicated that there might be a second species of *Trichuris* in arvicoline rodents.

The analysis of ITS2 sequences of *Trichuris* species from Argentina showed four main clades by ML, MP and BI methods corresponding with four different species regardless of the geographical origin and host species: *Trichuris navonae*, *T. pardinasi*, *Trichuris* sp. and *T. bainae* n. sp. These four species from sigmodontine rodents clustered together and separated from *Trichuris* species isolated from murine and arvicoline rodents (outgroup). Different genetic lineages were found among *Trichuris* species from sigmodontine rodents, which supported the proposal of a new species.

The host distribution of the studied species showed correspondence with different tribes included in Sigmodontinae rodents such as, Clade 1-Akodontini; Clade 2- Oryzomyini, Clade 4-Phyllotini. *Trichuris navonae* (Clade 1) is a parasite of *Akodon montensis*, one of the most abundant host species present in a wide geographical distribution from the Atlantic forest and cerrado in Brazil, Paraguay and Argentina [59]–[63]. Also, *T. navonae* is present in another sympatric host species, *Thaptomys nigrita*, which is considered rare (not easy to capture) and not abundant (not caught in numbers) [64], [65]. Both rodents occur in the same microhabitat, living in primary and secondary forests [66]. The new species *Trichuris bainae* n. sp. (Clade 2) is a parasite of *Sooretamys angouya,* a rodent with sympatric distribution with the two akodontines mentioned above, although this species uses trees and ground more frequently [64], [67]–[69]. This rodent is considered common (easy to capture) but not abundant (not caught in numbers) [64]. *Trichuris pardinasi* (Clade 3) is a parasite of *Phyllotis bonariensis* and *P. xanthopygus*, both abundant species which are found in a wide variety of habitats, but these are restricted mainly to rocky outcrops [70], [71]. *Phyllotis bonariensis* is distributed only in Sierra de la Ventana, southeast of Buenos Aires province (Argentina) [72], [73] while *P. xanthopygus* has a wide distribution, along the Andes from west central Peru to Santa Cruz Province (Argentina) and the adjacent Magellan Region of Chile [74], [75]. The population of *P. bonariensis* from Sierra de la Ventana was originally cited as an endemic species by Crespo [72], Reig [76], Galliari et al. [77], and Musser and Carleton [74]. Later, the populations of *P. xanthopygus* were considered as stated in Pardiñas et al. [78]. Currently, there is no solid evidence available to justify this second proposal and specific status [79]. In this paper we follow the first taxonomic proposal. However, Sierras de Córdoba and Sierra de la Ventana are areas considered faunistic islands that share a considerable number of species and subspecies, i.e., molluscs, insects, amphibians [80], as well as *T. pardinasi*.

In this study, three clades showed different levels of host specificity. Clade 1 and 2 indicated the presence of different species of *Trichuris* in the same biome, but with each species associated with a different host tribe and ecological habits. In addition, these species of whipworm follow their hosts along their geographical distribution (e.g. *T. bainae* in *S. angouya* from Misiones and Formosa provinces). Clade 4 indicated the presence of the same species of *Trichuris* in two congener host species in two disjunct areas; showing a probably specificity at the generic level of host (or specific level if futures studies confirm that *P. bonariensis* and *P. xanthopygus* are conspecific).

Although other closely related species of *Trichuris* spp. from Sigmodontinae, such as *T. laevitestis* from *Akodon azarae* (Fischer, 1829), *Scapteromys aquaticus* Thomas, 1920 and *Necromys lasiurus* (Lund, 1840) [3]; and *T. travassosi* from *Oligoryzomys nigripes* (Olfers, 1818) [6] were not studied here, it is possible that the correspondence between parasite-tribe host is maintained, even though probably the level of host specificity could be different in agree with the ecology of the species host and the history of the areas where the hosts lives [81].

The understanding of the phylogeography of these nematodes would be improved by the study of a larger number of specimens and integrating biogeographic information from potential hosts. For example, *Trichuris* spp. in Clade 3 consisted of only one specimen and it was not possible to provide any hypothesis about its host and geographical distribution.

This study highlights the importance of an integrated study of *Trichuris* spp., allowing a more complete understanding of the taxonomy, host and geographical distribution, and biology of whipworms.
